# A Facile and General Approach to Enhance Water Resistance of Metal-Organic Frameworks by the Post-Modification with Aminopropyltriethoxylsilane

**DOI:** 10.3390/nano12071134

**Published:** 2022-03-29

**Authors:** Jianmei Gu, Jianquan Li, Qingyu Ma

**Affiliations:** School of Materials Science and Engineering, University of Jinan, Jinan 250022, China; jianmeigu2022@163.com (J.G.); mse_lijq@ujn.edu.cn (J.L.)

**Keywords:** metal-organic framework (MOF), water resistance, post-modification, aminopropyltriethoxylsilane

## Abstract

The water sensitivity of metal-organic frameworks (MOFs) as a common and crucial issue has greatly hindered their practical applications. Here, we present a facile and general approach to improve the water resistance of a typical MOF, i.e., CuBTC [Cu_3_(BTC)_2_(H_2_O)_3_]_n_ (BTC = benzene-1,3,5-tricarboxylate) using a post-modification reaction with aminopropyltriethoxylsilane (APTES) at room temperature. The afforded material is denoted as CuBTC@APTES. Various spectroscopic methods reveal that the organosilicon linkers have been successfully grafted onto CuBTC by electrostatic attraction between acid and base groups and without affecting the original coordination mode and basic structure. Compared with CuBTC, the water stability of CuBTC@APTES was significantly improved. The pristine CuBTC almost lost all its crystallinity, morphology and pore structure after 3-day treatment in water, while CuBTC@APTES is able to retain its main crystal structure and basic porosity after the same treatment. This finding can be explained by the successful introduction of the organosilicon molecular overlayer on the periphery of CuBTC to slow down the destruction of weak metal coordination bonds by water molecules, thus improving the water stability of CuBTC. The solution of water sensitivity provides more opportunities for the practical applications of CuBTC, such as aqueous phase catalysis and gas separation in humid environments. This simple approach can certainly be expanded to improve the water resistance of other carboxylate-containing ligand-based MOFs.

## 1. Introduction

Metal-organic frameworks (MOFs) are a well-known class of porous crystals, which are constructed by the coordination assembly of metal ions (or metal clusters) with organic ligands [1,2,3,4]. They have found extensive applications in gas adsorption and separation, catalysis, and sensor, by virtue of their ultrahigh surface areas, pore volumes and tunable properties [5,6,7,8,9,10,11,12,13]. Furthermore, CuBTC [Cu_3_(BTC)_2_(H_2_O)_3_]_n_ (BTC = benzene-1,3,5-tricarboxylate) (also known as HKUST-1) is one of the most studied MOFs [14,15,16], and is composed of dimeric cupric tetracarboxylate units, constructed by Cu_2_ paddlewheel clusters linked by BTC^3−^ ligands [17,18,19,20,21,22,23]. Attributed to its structural characteristics, CuBTC not only has a high specific surface area and pore structure, but also has abundant active centers, making it suited to potential applications in gas adsorption, catalysis and other fields [24]. In these practical applications, the presence of water is often unavoidable [25]. However, stability is still considered a major barrier impeding its wide use in various applications [26,27,28,29,30,31]. For example, when HKUST-1 was utilized as a catalyst in the aqueous reaction of benzaldehyde and *o*-phenylenediamine, the yield of benzimidazole was 94%. However, the yield reduced to 63% after three cycles, while the BET surface areas of HKUST-1 decreased from 1451 to 135 m^2^/g. In contrast, after the hydrophobic modification, the catalytic activity of HKUST-1-P remained unchanged in three successive reactions, and the yield increase to 99%. Additionally, the BET surface areas decreased slightly [32]. Therefore, pure HKUST-1 is not suitable for aqueous catalytic reactions and it is necessary to improve the water stability of HKUST-1 for the application of green and sustainable water as the solvent of catalytic reactions. Barbara et al. studied the interaction between biologically organic molecules (e.g., caffeine, urea and glycine) and the uncoordinated copper centers of CuBTC. In any case, H_2_O is always competing for open-adsorption positions. In addition, due to the instability of CuBTC to water, toxic copper may be released into the body during frameworks decomposition, thereby limiting its medical applications [33]. On the one hand, CuBTC is extremely hydrophilic and can bind water molecules instantaneously, even in air [34,35,36]. Metal-ligand coordinative bonds are weak and susceptible to water molecules [37]. The coordinative bonds are gradually replaced by water molecules, resulting in the destruction of MOFs structures [38,39]. It has been suggested that water molecules must aggregate around the second building units (SBUs) before the occurrence of CuBTC degradation [40]. Therefore, a method by which to slow down the damage of water molecules aggregation around SBUs to coordination bonds is urgently needed.

Currently, there are two main strategies to achieve MOFs with high water stability [41]. One is to use metal ions with strong coordination bonds or hydrophobic ligands to directly synthesize MOFs with a high stability [37,42,43]. The other is to improve the stability of the existing MOFs using various methods such as post-synthetic modification [44,45,46,47]. Considering that the reasonable strategies for preparing stable MOFs by de novo synthesis are still limited, the stability improvement of the existing MOFs has attracted specific attention. For example, in the case of glycine-doped CuBTC, a post-synthetic modification (PSM) method has been reported to expand its practical applications [48]. Unfortunately, the porosity of the modified Gly-CuBTC has decreased. Zhang et al. proposed a vapor deposition technique by which to modify hydrophobic polydimethylsiloxane (PDMS) on the surface of MOF materials, and the water stability was significantly improved [49]. Although the pore characteristics of CuBTC basically remain unchanged, the treatment methods are complicated and tedious. Ding et al. developed a surface polymerization technique, in which monomers polymerized on the outer surface of CuBTC to form a hydrophobic layer, which improves the moisture resistance of the product [32]. This inspired us to develop a more facile and new approach to improve the water stability of CuBTC.

Herein, we present a facile and general approach to improve the water stability of CuBTC using a post-synthetic modification of CuBTC with a low-cost and commercially available silane agent, aminopropyltriethoxylsilane (APTES), yielding CuBTC@APTES. The water resistance performances of CuBTC and CuBTC@APTES were evaluated by immersing them in water. The structures of the materials before and after treatment were characterized by X-ray diffraction, scanning electron microscopy and N_2_ adsorption measurements, so as to evaluate the water stability. It was found that this strategy can significantly enhance the water resistance of CuBTC.

## 2. Materials and Methods

### 2.1. Materials

Copper nitrate trihydrate [Cu(NO_3_)_2_·3H_2_O, 99%], 1,3,5-Benzenetricarboxylic acid (BTC, 98%), aminopropyltriethoxylsilane (APTES, 99%), and dichloromethane (99.5%) were purchased from Shanghai Aladdin Biochemical Technology Co., Ltd. (Shanghai, China). Methanol (absolute dry) was obtained from Sinopharm Chemical Reagent Co., Ltd. (Shanghai, China). All materials were used as received without further purification.

### 2.2. Synthesis of CuBTC

CuBTC was synthesized according to the procedure reported by Wu et al. [32,50,51]. According to typical procedure, BTC (0.875 g, 4.16 mmol) was dissolved in a 50 mL absolute methanol under ultrasonication for 2 h. To this solution, a mixture of Cu(NO_3_)_2_·3H_2_O (1.82 g, 7.53 mmol) and absolute methanol (50 mL) was transferred into the trimesic acid solution. After that, the solution was kept for 2 h at room temperature until CuBTC precipitation was formed. The blue precipitation was separated by centrifugation and washed with methanol twice. Finally, the blue powder of CuBTC was obtained by drying overnight under vacuum at room temperature (0.25 g, yield: 18.2%).

### 2.3. Preparation of CuBTC@APTES

APTES functionalized CuBTC (denoted as CuBTC@APTES) were prepared by the following procedure. First, CuBTC (0.328 g, 0.5 mmol) was uniformly dispersed in 10 mL dichloromethane under ultrasonication for 10 min and APTES was added to the solution drop by drop. The resultant mixture was stirred for 24 h at room temperature. Additionally, the functionalized products were then separated by centrifugation and washed with dichloromethane three times to remove the unreacted APTES. Finally, the products were dried overnight under vacuum at room temperature. Three kinds of CuBTC@APTES samples were synthesized using 0.111 g (0.5 mmol), 0.221 g (1 mmol) and 0.332 g (1.5 mmol) of APTES loading in the pristine CuBTC. Accordingly, the functionalized materials have been denoted as CuBTC@APTES-1, CuBTC@APTES-2, CuBTC@APTES-3 in the following sections corresponding to Cu/APTES ratios of 3, 1.5 and 1, respectively.

### 2.4. Characterization

X-ray diffraction (XRD) was performed using a Bruker D8 Advance X-ray diffractometer (Billerica, MA, USA) (Cu Kα radiation, 40 kV, 40 mA, λ = 1.5406 Å) at room temperature with a step-scan mode (0.02° per step), a scan speed of 2°/min and an angular range of 5° < 2θ < 50°. Morphological features of the samples were observed using scanning electron microscopy (SEM) which was carried out using a Regulus 8100 (Tokyo, Japan) with a high voltage mode of 20 kV. Before scanning the sample with SEM, an ultrathin gold coating of the sample was carried out using the Cressington sputter coater (Watford, United Kingdom) in order to create a conductive layer on the surface of materials to inhibit their charging. Fourier Transform Infrared (FT-IR) spectra were measured using a Nicolet iS10 spectrometer produced by Thermo Scientific (Waltham, MA, USA), with KBr pellets in the 4000–400 cm^−1^ wavenumber region. ^1^H NMR spectra were recorded using Avance III 400 MHz spectrometers (Karlsruhe, Germany). The X-ray photoelectron spectroscopy (XPS) analyses were carried out using the ESCALAB Xi+ XPS spectrometer produced by Thermo Fisher Scientific (Waltham, MA, USA). The contents total of copper and silicon were quantified using an inductively coupled plasma optical emission spectrometer (ICP-OES) with an Agilent 720ES (Santa Clara, CA, USA). The thermogravimetric analysis (TGA) was performed on a TGA5500 thermogravimetric analyzer (Columbus, OH, USA), with heating temperature ranging from 30 to 800 °C at a rate of 10 °C min^−1^ in nitrogen atmosphere. The water contact angle measurement was measured using an JC2000D2G contact angle meter (Shanghai, China) at ambient conditions. The powder was processed using a tablet press using a pressure of 2 MPa. Sessile water drops of 3 μL were used. The nitrogen adsorption–desorption isotherms were measured to determine the pore textural properties using a Micromeritics ASAP 2460 (Norcross, GA, USA) at 77 K, equipped with commercial software (MicroActive for ASAP 2460) for calculation and analysis. Prior to the measurement of adsorption isotherms, the samples were outgassed under vacuum at 120 °C for 8 h. The Brunauer-Emmett-Teller (BET) method was applied to determine specific surface areas of materials.

## 3. Results

### 3.1. Synthesis and Characterization of CuBTC and CuBTC@APTES

**Scheme 1:**
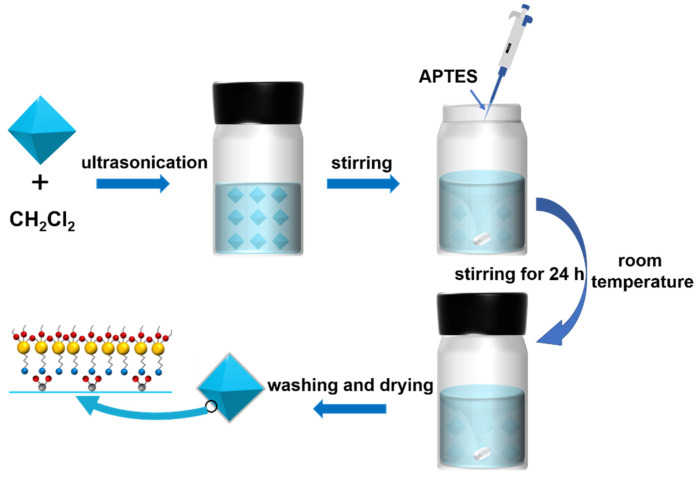
Schematic illustration for the process of CuBTC@APTES preparation.

**Figure 1:**
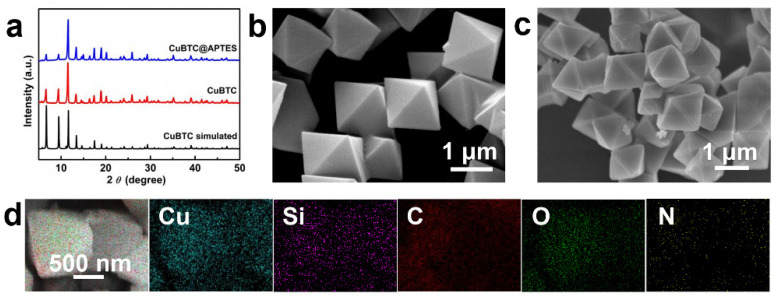
(**a**) XRD patterns of CuBTC and CuBTC@APTES; (**b**,**c**) SEM images of CuBTC and CuBTC@APTES; (**d**) EDX mapping of CuBTC@APTES.

There exist some uncoordinated carboxylic acid groups on the surface of CuBTC, which can interact with the amino groups on APTES by electrostatic attraction. CuBTC@APTES were prepared following a simple method (Scheme 1). CuBTC was uniformly dispersed in dichloromethane under ultrasonication and APTES were added drop by drop. The mixture was stirred at room temperature for 24 h. After washing and drying, the final product was obtained. As shown in Figure 1, the structure, morphology and distribution of the elements of the samples were monitored using X-ray diffraction (XRD), scanning electron microscopy (SEM) and EDX mapping. In the XRD pattern of CuBTC@APTES, the main characteristic peaks are consistent with that of CuBTC, indicating the retention of the crystalline lattice structure and well-inheritance of the original crystalline structure after the post functionalization of CuBTC (Figure 1a). Pristine CuBTC crystals exhibit a regular octahedral shape with an average size of 1 μm as shown in SEM image (Figure 1b). As shown in Figure 1c, the size of the CuBTC@APTES crystals is similar to that of CuBTC. Additionally, the octahedron surface is relatively rough because of the introduction of APTES. EDX mapping was also carried out to explore the distribution state of elements in the CuBTC@APTES. The presence and uniform distributions of Si and N indicate that APTES was uniformly modified on the CuBTC surface (Figure 1d).

**Figure 2:**
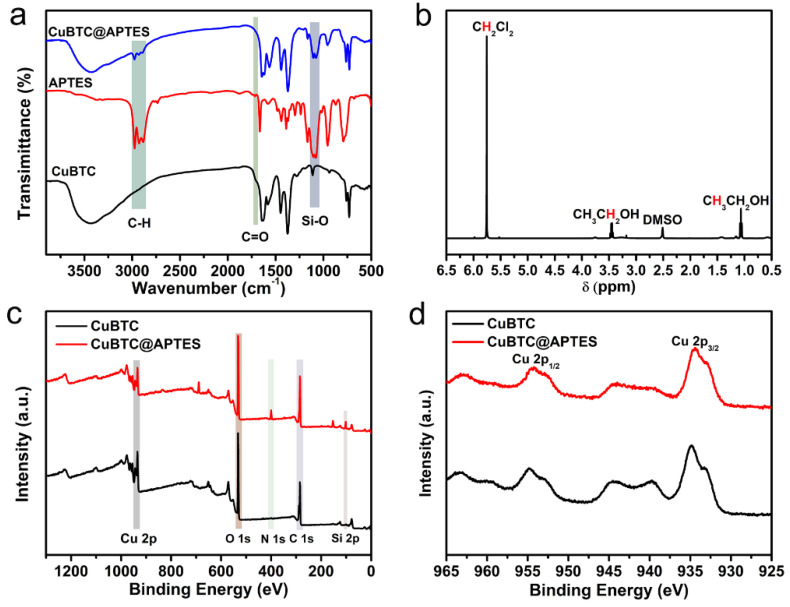
(**a**) FT-IR spectra of CuBTC, APTES and CuBTC@APTES; (**b**) ^1^H NMR spectra of the reaction solution of CuBTC@APTES; (**c**) XPS spectra of CuBTC and CuBTC@APTES; (**d**) high-resolution XPS spectra for Cu2p in CuBTC and CuBTC@APTES.

To achieve more internal structure information and understand the modification details, the structures were further investigated. The FT-IR spectra of CuBTC, APTES and CuBTC@APTES were measured (Figure 2a). For the CuBTC particles, there are four characteristic peaks at 1636, 1564, 1440 and 1371 cm^−1^, assigned to the -COO- vibration peak of the BTC ligand. These peaks are also found from CuBTC@APTES, suggesting that the coordination in Cu_2_C_4_O_8_ cages of the CuBTC frameworks were unchanged after modification. The peak at 1708 cm^−1^ in the FT-IR spectra of CuBTC is attributed to the carboxylic group, while it disappears in CuBTC@APTES. This finding reveals that the carboxylic groups interacted with amino groups by protonation, leading to the deprotonation of the carboxylic acid. [52]. Meanwhile, the adsorption bands at 2880–2970 cm^−1^ associated with the C-H stretching vibration peaks from APTES was also observed in CuBTC@APTES. More importantly, the peak induced by the stretching vibration of Si-O appears at 1075 cm^−1^, indicating that the organosilicon linkers were successfully grafted on the surface of CuBTC. Unfortunately, solid nuclear magnetism cannot obtain good results due to the strong paramagnetic relaxation caused by the presence of Cu^2+^ in the frameworks [52]. For this reason, the reaction solution of CuBTC@APTES was monitored by ^1^H NMR (Figure 2b). The proton peaks at 1.03 and 3.49 ppm derive from ethanol. Considering the acidic feature of CuBTC, APTES is easy to hydrolyze to produce ethanol and silanol. On the one hand, the amino group on APTES is connected to the carboxylic acid group on the surface of CuBTC by electrostatic attraction. On the other hand, the ethoxyl group of APTES reacts with free water in CuBTC in an acidic environment to hydrolyze ethanol and silanol, which can be further condensed to Si-O-Si bonds so that the organosilicon molecule is firmly modified on the pristine CuBTC (Scheme 1) [32,53]. The X-ray photoelectron spectroscopy (XPS) measurements for CuBTC and CuBTC@APTES were also studied. As expected, CuBTC contains elements Cu, O, and C, while CuBTC@APTES contains Cu, O, N, C, and Si (Figure 2c). This finding is consistent with EDX mapping results. The high-resolution XPS spectra of Cu2p in CuBTC and CuBTC@APTES were shown in Figure 2d. CuBTC shows an intense Cu2p3/2 peak at a binding energy of 934.2 eV and Cu2p1/2 peak at a binding energy of 954 eV, which is an intrinsic Cu(II) state within the structure. The binding energy of Cu2p in CuBTC@APTES does not show any obvious change, implying that the oxidation state of Cu remains the same before and after APTES modification process. These results reveal that the binding of CuBTC and APTES does not depend on the coordination interaction between the Cu atoms from CuBTC and the polymer, which is consistent with the previous results.

**Figure 3:**
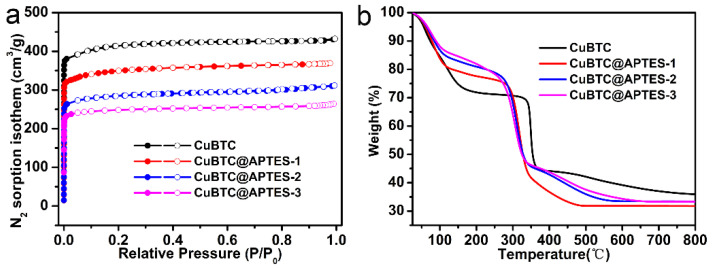
(**a**) N_2_ adsorption–desorption isotherms of CuBTC and CuBTC@APTES; (**b**) TGA curves of CuBTC and CuBTC@APTES.

In order to quantify the contents of total copper and silicon in CuBTC@APTES samples, different amounts of APTES were used and the resulting samples were characterized by ICP-OES (Table S1 from the Supplementary Materials). The mole ratio of Cu/Si gradually decreases with the increment of APTES amount. However, the overall proportion is higher than the theoretical values. This phenomenon may be due to the fact that APTES did not fully react with CuBTC and the depletion of Si elements during digestion and testing. Nitrogen adsorption–desorption isotherms at 77 K were also used to evaluate the porosity of the materials before and after modification. As shown in Figure 3a, all samples show typical type I isotherms, revealing their predominate microporosity of the frameworks. The BET surface area of the CuBTC is measured at 1644 m^2^/g, which is similar to previously reported data [49]. As expected, with increasing the amount of APTES, the BET surface areas of CuBTC@APTES decreases to 1381 m^2^/g (CuBTC@APTES-1), 1135 m^2^/g (CuBTC@APTES-2) and 1010 m^2^/g (CuBTC@APTES-3) (Table S2). Such decrease suggests that the original porous structure of the matrix has been partially blocked after APTES modification. In addition, the introduction of excessive APTES will have a deeper impact on the porous structure of CuBTC@APTES samples, which is not conducive to the adsorption application. TGA was used to evaluate the thermal behaviours of these materials in nitrogen atmosphere (Figure 3b). CuBTC has two well-separated weightlessness steps, namely removal of solvent and water molecules (30–180 °C) and decomposition of skeleton (>330 °C), with no significant weight loss between the two processes. For modified materials, there is an additional weightlessness stage (180–240 °C). This may be attributed to the decomposition of the grafted organic groups. Additionally, with the increment of APTES amount, the weight loss phenomenon is more obvious because of the increment of organic content. In addition, the final decomposition temperature of the modified materials decreases due to the addition of organic materials and higher dosage lead to lower decomposition temperature.

**Figure 4:**
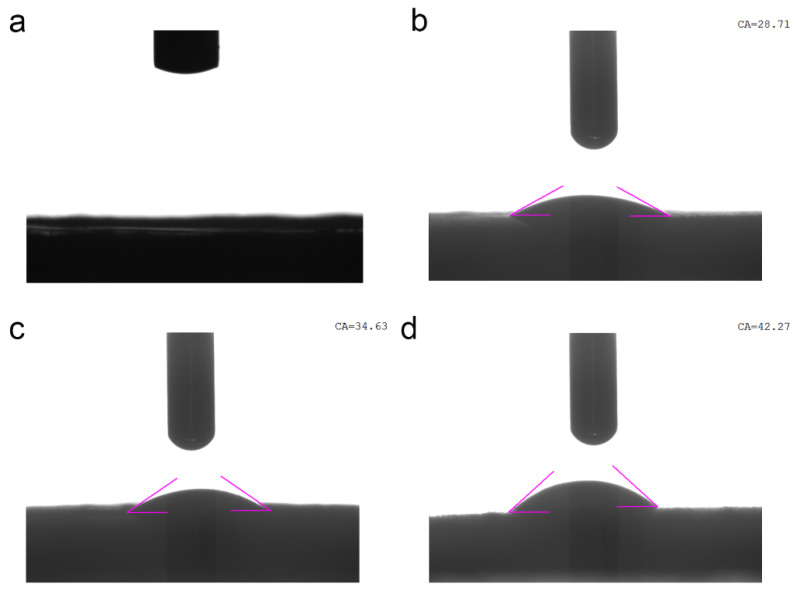
Static contact angles of water on (**a**) CuBTC; (**b**) CuBTC@APTES-1; (**c**) CuBTC@APTES-2; (**d**) CuBTC@APTES-3.

The static contact angle (CA) measurements were also carried out to study the surface wettability of CuBTC and CuBTC@APTES (Figure 4). The water CA of CuBTC is close to ~0°, indicating its intensely hydrophilic property (Figure 4a). Water droplets diffuse easily on CuBTC and are quickly absorbed by it. Compared to CuBTC, the water CAs for CuBTC@APTES-1, CuBTC@APTES-2, CuBTC@APTES-3 are 28.7, 34.6, and 42.3° (Figure 4b,c). The increased water contact angles can be attributed to the introduction of the coating on the surface. To some extent, the introduction of organosilicon molecular layer can ameliorate the extreme hydrophilic characteristics of CuBTC so as to reduce the attack of water molecules on CuBTC and protect it.

### 3.2. Water Stability of CuBTC and CuBTC@APTES

To evaluate the water stability of pristine and modified CuBTC samples, all samples were immersed in water at room temperature for 24 h. The XRD patterns of CuBTC and CuBTC@APTES after 24 h immersion in water are displayed (Figure S1). It shows that the original diffraction peaks of CuBTC disappeared after 1 day of exposure to water, and thus the structural integrity was obviously lost. However, the main characteristic peaks of each proportion of CuBTC@APTES samples were unchanged after the same water treatment. This transformation indicates that the water stability of CuBTC@APTES significantly improved after modification. In the subsequent experimental treatment, we soaked the modified samples at a higher temperature of 50 °C for 24 and 48 h, respectively, to study their performance (Figure S2). The XRD results show that the diffraction peaks of CuBTC@APTES-1 and CuBTC@APTES-3 changed after 24 h water immersion. The CuBTC@APTES-2 sample still retained its original crystal shape even after 48 h of water treatment. Therefore, the comparison of the three samples shows that CuBTC@APTES-2 has the best water-resistance performance. In addition, the stability of the CuBTC@APTES in different solvents was also studied. The samples were immersed in different solvents for 24 h and the XRD comparison results demonstrate that the samples can remain stable in various solvents, such as ethanol, methanol, cyclohexane, DMF, ethyl acetate, and petroleum ether (Figure S3).

**Figure 5:**
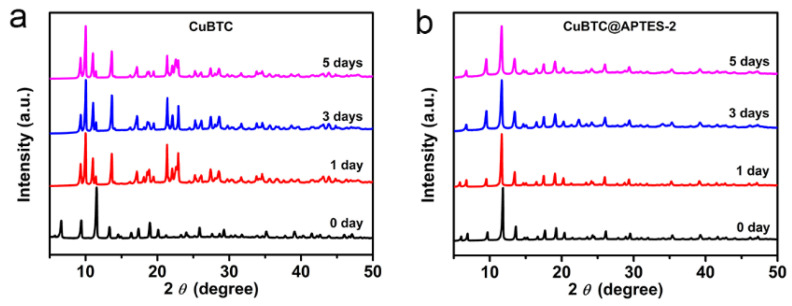
XRD patterns of samples after submersion in water (**a**) CuBTC; (**b**) CuBTC@APTES-2.

Based these results, it can be concluded that CuBTC@APTES-2 sample has the best water stability. Therefore, its water stability was further estimated by extending the immersion time in water for 0, 1, 3 and 5 days at room temperature. After drying, the samples were tested by XRD, SEM and N_2_ adsorption measurements. The XRD patterns of CuBTC and CuBTC@APTES-2 after immersion in water are displayed (Figure 5). There is a significant loss in the structural integrity for CuBTC after 1 day’s exposure in water, while the main characteristic peaks of CuBTC@APTES-2 remain unchanged even after 5 days’ exposure. In other words, the modification of APTES towards CuBTC greatly enhanced its framework stability.

**Figure 6:**
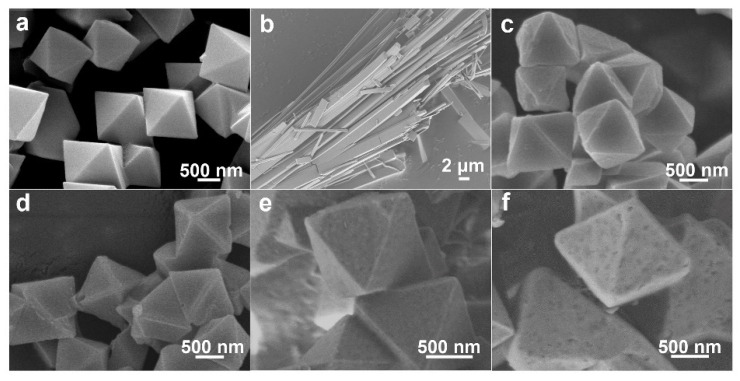
(**a**,**b**) SEM images of CuBTC (**a**) and CuBTC after submersion in water for 1 day (**b**); (**c**–**f**) SEM images of CuBTC@APTES-2 (**c**) and CuBTC@APTES-2 after submersion in water for 1 day (**d**), 3 days (**e**), and 5 days (**f**).

SEM images can also prove the stability. For CuBTC, the original octahedral morphology partly disappears and becomes rod-shaped (Figure 6a,b) after being subject to the aqueous environment for 1 day. The low dimensionality may be caused by the ligation of three aqua groups per square-pyramidal Cu^2+^ ion [15]. In contrast, the CuBTC@APTES-2 particles treated in water for 1 day can well preserve the octahedral shape of the sample (Figure 6c,d). After 3 days, the surface of CuBTC@APTES-2 remains essentially unchanged (Figure 6e). It is worthy of note that the material can still remain the octahedral shape even after five days of water treatment, although some pores appeared on the surface (Figure 6f). These results indicate that the modification of APTES can repel water molecules to attack Cu^2+^, thereby improving the water stability of CuBTC. In other words, the introduction of the organosilicon molecular layer can slow down the contact of water molecules with Cu^2+^ and further hinder the destruction of Cu-O coordination bonds by water molecules. Therefore, more time is required for water molecules to bond with Cu^2+^ and further destroy the entire structure.

**Figure 7:**
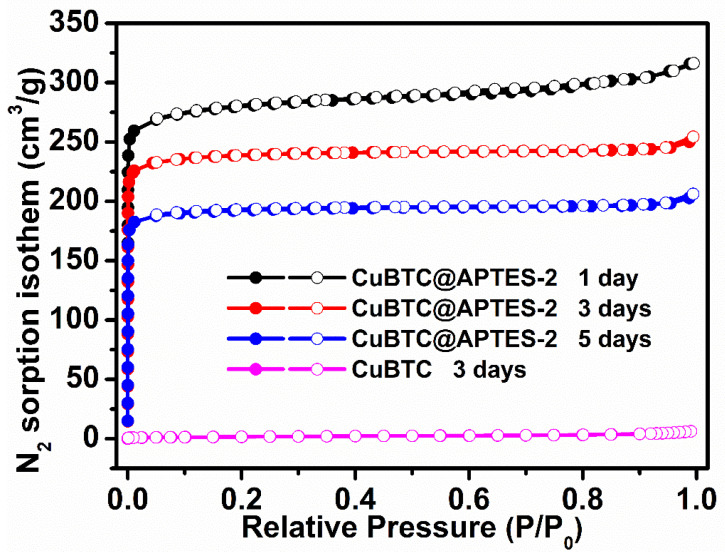
N_2_ adsorption–desorption isotherms at 77 K of CuBTC and CuBTC@APTES-2 after submersion in water for several days.

The enhanced water stability of CuBTC under this simple strategy can be also evidenced by the porosity change. The porous properties of all samples before and after the water treatment were evaluated by N_2_ adsorption–desorption isotherms at 77 K (Figure 7). Upon immersion in water for 3 days, the BET surface area of CuBTC significantly reduced from 1644 to 37 m^2^/g, demonstrating that the porous structures of the original frameworks completely collapsed. In contrast, the BET surface area of CuBTC@APTES-2 can remain relatively high. After 1 day’s exposure in water, the BET surface areas of CuBTC@APTES-2 were measured as 1112 m^2^/g and thus remained essentially unchanged in comparison to the untreated CuBTC@APTES-2 (1135 m^2^/g). After immersion in water for 3 days, the BET surface area was as high as 967 m^2^/g (~13% drop). Even after 5 days water treatment, the BET surface area was still as high as 787 m^2^/g. These findings clearly indicate that there is almost no structural damage to the CuBTC@APTES-2 sample during the water treatment because the copper centers protected by the organosilicon molecules remain intact in the water molecules. These results reveal that the developed method can slow down the destruction of the frameworks by water molecules and efficiently preserve structural integrity.

## 4. Conclusions

In conclusion, a facile and general method to enhance the water stability of CuBTC has been successfully developed by modifying CuBTC with APTES. The structures and morphology of the formed CuBTC@APTES were fully characterized by FT-IR, XRD, and SEM. The results indicate the successful grafting of organosilicon molecules on the periphery of CuBTC without affecting the original coordination mode and basic structure. In other word, CuBTC@APTES can well inherit the original crystalline structure and morphology of CuBTC after the modification treatment. The water-resistance performances of CuBTC and CuBTC@APTES were evaluated by immersion in water. As the sample with the best water stability among the three samples, the CuBTC@APTES-2 sample can retain its crystal structure even after 48 h of water treatment at a temperature of 50 °C. After a longer period of water treatment at room temperature, CuBTC@APTES-2 generally maintains its crystal structure, morphology and surface, and still has better structural integrity, while CuBTC collapses quickly. This contrast occurs because the successful introduction of organic materials protects the weak metal coordination bonds from the damage of water molecules and thus improves the water stability of CuBTC@APTES. The improved stability of water provides more opportunities for the practical use of CuBTC in industry. In particular, the modified materials can be used in situations where water is present, such as aqueous phase catalysis and gas separation in humid environments. In short, the improved structural stability is expected to facilitate its use in harsher environments. In addition, based on the bonding effect of silane coupling agents, the class of molecules can be introduced into a variety of MOFs to customize different functions. The water stability of additional MOFs can be enhanced by this simple strategy.

## Data Availability

The data presented in this study are available on request from the corresponding author.

## Supplementary Materials

The following supporting information can be downloaded at: https://www.mdpi.com/article/10.3390/nano12071134/s1, Figure S1: XRD patterns of CuBTC and CuBTC@APTES after submersion in water for 24 h; Figure S2: XRD patterns of CuBTC@APTES after submersion in water of 50 °C; Figure S3: XRD patterns of CuBTC@APTES after submersion in different solvents for 24 h; Table S1: ICP-OES of CuBTC@APTES and the ratio of Cu/Si; Table S2: BET Surface area parameters of all samples before and after water treatment.
