# Supplementary material for: A Facile and General Approach to Enhance Water Resistance of Metal-Organic Frameworks by the Post-Modification with Aminopropyltriethoxylsilane
Source: Nanomaterials (Basel). 2022 Mar 29;12(7):1134. doi: 10.3390/nano12071134 (PMC9000530; doi:10.3390/nano12071134)
Supplement: Supplementary file 1 [file nanomaterials-12-01134-s001.zip › nanomaterials-1652828-supplementary.pdf]

Supplementary Materials

A Facile and General Approach to Enhance Water Resistance of Metal-Organic Frameworks by the Post-Modification with Aminopropyltriethoxylsilane

Jianmei Gu, Jianquan Li and Qingyu Ma *

School of Materials Science and Engineering, University of Jinan, Jinan 250022, China; jianmeigu2022@163.com (J.G.); mse_lijq@ujn.edu.cn (J.L.)

* Correspondence: mse_maqy@ujn.edu.cn

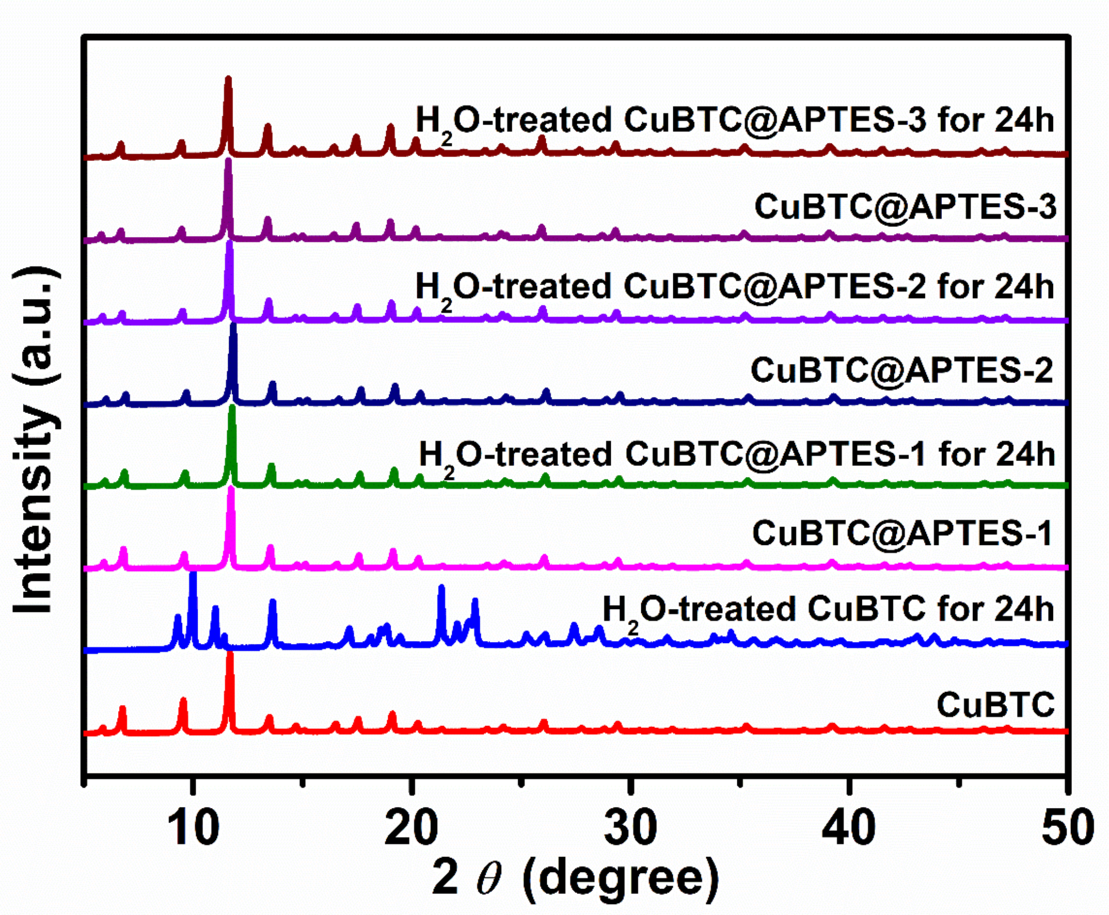

Figure S1. XRD patterns of CuBTC and CuBTC@APTES after submersion in water for 24 h.

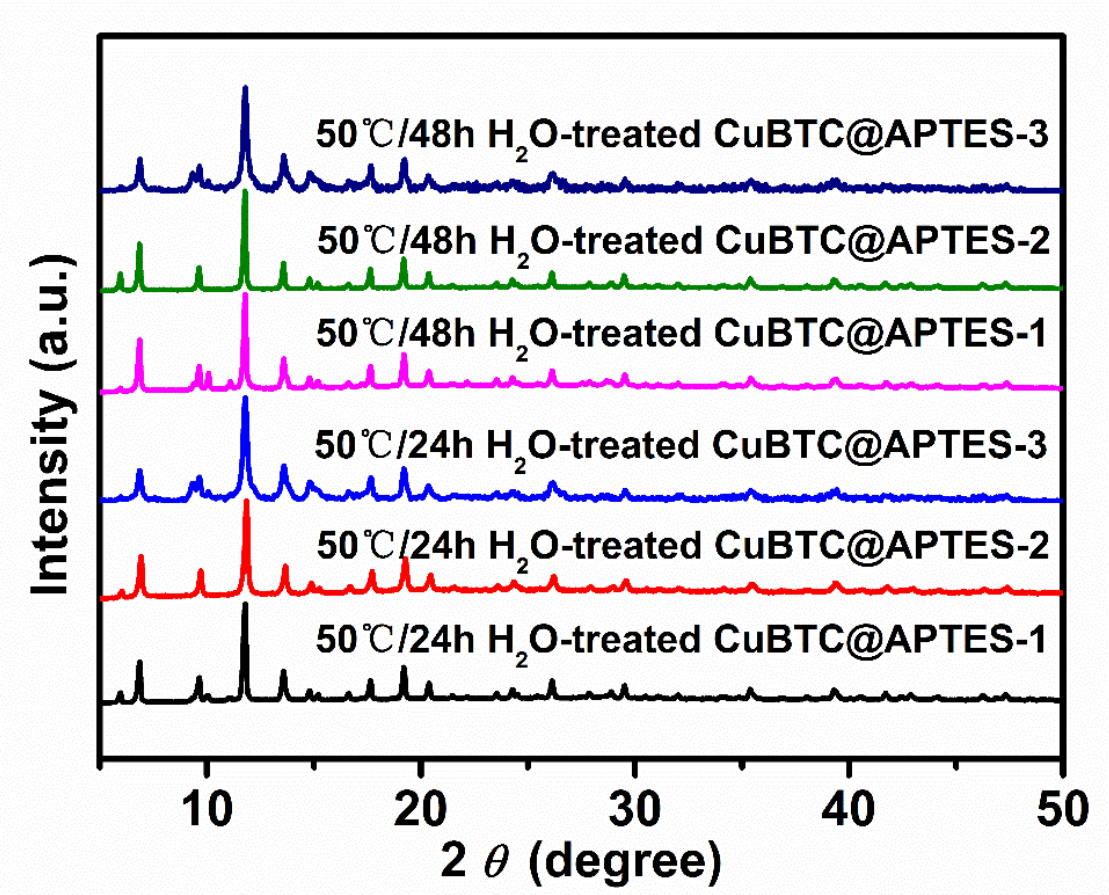

Figure S2. XRD patterns of CuBTC@APTES after submersion in water of 50 °C.

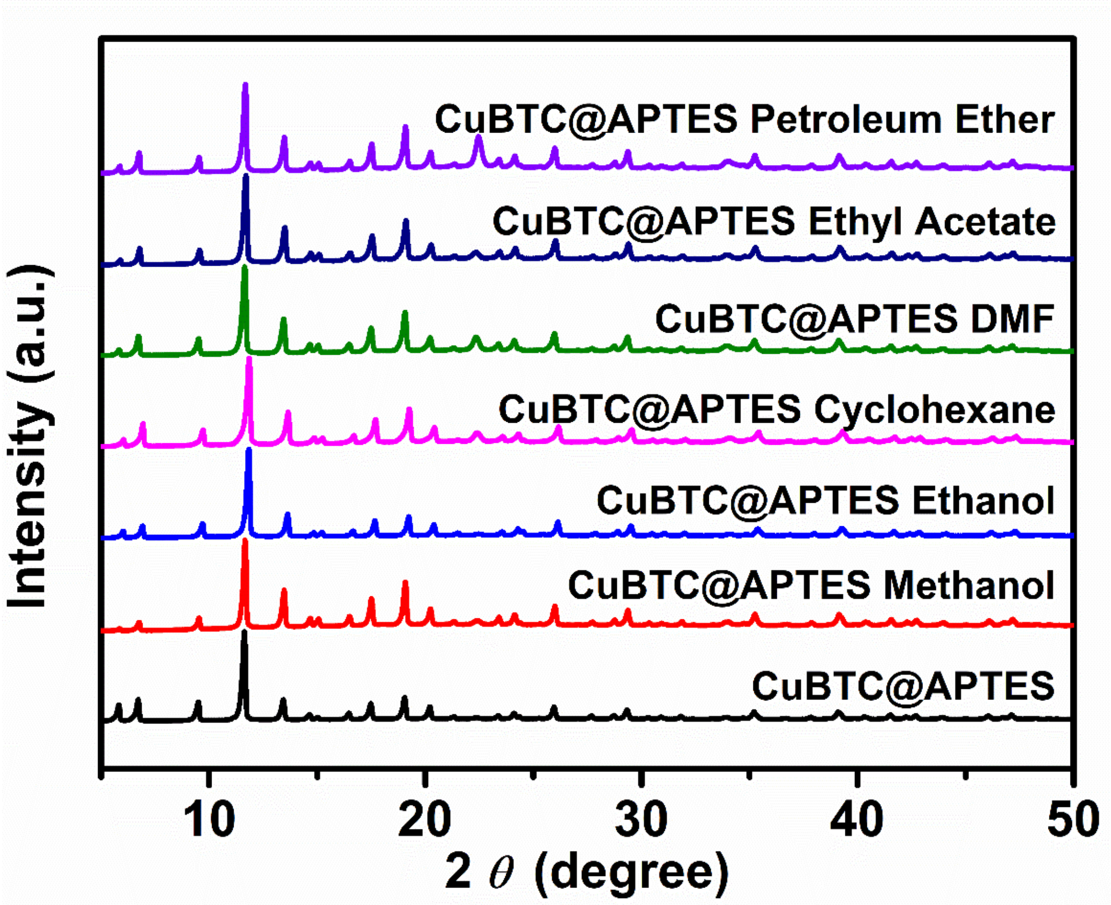

Figure S3. XRD patterns of CuBTC@APTES after submersion in different solvents for 24 h.

Table S1. ICP-OES of CuBTC@APTES and the ratio of Cu/Si.

CuBTC@APTES	Cu/wt%	Si/wt%	Cu/Si
CuBTC@APTES-1	21.68	1.63	5.88
CuBTC@APTES-2	20.99	1.75	5.33
CuBTC@APTES-3	19.55	2.83	3.05

Table S2. BET Surface area parameters of all samples before and after water treatment.

<table>
<tr><th>Sample</th><th>S_{BET} (m^2/g)</th><th>S_{BET} (m^2/g) after water treatment</th></tr>
<tr><td>CuBTC</td><td>1644</td><td>37 (3 days)</td></tr>
<tr><td>CuBTC@APTES-1</td><td>1381</td><td rowspan="3">1112 (1 day), 967 (3 days), 787 (5 days)</td></tr>
<tr><td>CuBTC@APTES-2</td><td>1135</td></tr>
<tr><td>CuBTC@APTES-3</td><td>1010</td></tr>
</table>
